# Pro-Inflammatory and Immunological Profile of Dogs with Myxomatous Mitral Valve Disease

**DOI:** 10.3390/vetsci9070326

**Published:** 2022-06-28

**Authors:** Diego Piantedosi, Nadia Musco, Anna Teresa Palatucci, Flavia Carriero, Valentina Rubino, Francesco Pizzo, Saad Nasir, Giuseppe Molinaro, Giuseppina Ruggiero, Giuseppe Terrazzano, Pietro Lombardi, Laura Cortese

**Affiliations:** 1 Department of Veterinary Medicine and Animal Productions, University of Napoli Federico II, 80137 Naples, Italy; nadia.musco@unina.it (N.M.); francesco.pizzo2@unina.it (F.P.); saadnasir1992@gmail.com (S.N.); giuseppe.molinaro@unina.it (G.M.); pilombar@unina.it (P.L.); lcortese@unina.it (L.C.); 2 Department of Sciences, University of Basilicata, 85100 Potenza, Italy; anna.palatucci@unibas.it (A.T.P.); flavia.carriero@unibas.it (F.C.); giuseppe.terrazzano@unibas.it (G.T.); 3 Department of Translational Medical Sciences, University of Napoli Federico II, 80131 Naples, Italy; valentina.rubino@unina.it (V.R.); giruggie@unina.it (G.R.)

**Keywords:** dog, myxomatous mitral valve disease, cytokines, immunophenotype, Treg cells

## Abstract

**Simple Summary:**

Myxomatous mitral valve disease (MMVD) is the most commonly acquired cardiac disease in dogs and is responsible for congestive heart failure. In this research, some inflammatory, immunological, and echocardiographic parameters were evaluated in dogs affected by MMVD in order to assess the involvement of additional pathophysiological mechanisms during the disease. The main results revealed that inflammation parameters increased according to the severity of the disease and suggested that inflammatory activation may play an important role in cardiac remodeling associated with the progressive volumetric overload in MMVD. Also, a relative increase in Treg cells was detected, suggesting that they could represent a regulatory mechanism for limiting the inflammatory immune response.

**Abstract:**

Myxomatous mitral valve disease (MMVD) is a very frequently acquired cardiac disease in dog breeds and is responsible for congestive heart failure (CHF). The involvement of the immune system and pro-inflammatory cytokines in dogs with CHF due to mitral valve disease has not yet been extensively investigated. Here, we investigate the role of pro-inflammatory cytokines and the dysfunction of the immune system in dogs with different stages of severity through the blood assessment of CD4^+^FoxP3^+^regulatory T cells (Treg) cells, leptin, tumor necrosis factor (TNF)-α, interleukin (IL)-1β and IL-6 pro-inflammatory cytokines, and immunological and echocardiographic parameters. A total of 36 cardiopathic dogs, 14 females and 22 males, with MMVD were included. Mean age and body weight (BW) at the time of enrollment were 10.7 ± 2.77 years and 10.9 ± 6.69 kg, respectively. For the comparison of the pro-inflammatory and immunological parameters, two groups of healthy dogs were also established. Control group 1 consisted of young animals (n. 11; 6 females and 5 males), whose age and mean weight were 4.1 ± 0.82 years and 13.8 ± 4.30 kg, respectively. Control group 2 consisted of elderly dogs (n. 12; 6 females and 6 males), whose age and BW were 9.6 ± 0.98 years and 14.8 ± 6.15 kg, respectively. Of particular interest, an increase in Treg cells was observed in the cohort of MMVD dogs, as compared to the healthy dogs, as Treg cells are involved in the maintenance of peripheral tolerance, and they are involved in etiopathogenetic and pathophysiological mechanisms in the dog. On the other hand, TNF-α, IL-1β, and IL-6 significantly increased according to the severity of the disease in MMVD dogs. Furthermore, the positive correlation between IL-6 and the left ventricle diastolic volume suggests that inflammatory activation may be involved in cardiac remodeling associated with the progressive volumetric overload in MMVD.

## 1. Introduction

The presence of pro-inflammatory cytokines, such as TNF-α, IL-1, and IL-6, that may contribute to the pathogenesis of cardiac remodeling, and to systolic and diastolic dysfunction, is frequently described in human heart failure [1]. These pro-inflammatory cytokines could alter the phenotype and function of myocardial cells, suppress contractile function in cardiomyocytes, induce activation in macrophages, stimulate microvascular inflammation and dysfunction, and promote a matrix-degrading phenotype in fibroblasts. In addition, during chronic or congestive heart failure, the Renin-Angiotensin-Aldosterone System (RAAS) is activated. Angiotensin (AG) II and aldosterone are responsible for ventricular and renal remodeling over time through a pro-inflammatory and fibrosis-promoting action. An excess of aldosterone is a cardiovascular risk factor, potentially inducing vascular dysfunction and remodeling, increasing generation of reactive oxygen species (ROS) and inflammation, and activating the immune system [2]. Elevated levels of aldosterone (a phenomenon known as “aldosterone breakthrough”) have been found in patients treated with ACE inhibitors as the hormone can be produced alternatively or due to the existence of different isoforms of the angiotensin-converting enzyme (ACE). Also, for AG II, it is considered that it can be produced locally in the tissues (paracrine action) in patients treated with ACE inhibitors through alternative pathways (e.g., the chymase pathway). In kidney cells of human patients who have received kidney transplantation, it has been shown that aldosterone acts directly on T lymphocytes, modulating their activation and proliferation [3]. Additionally, a reduction in CD4^+^FoxP3^+^regulatory T cells (Treg cells) [4,5,6] was observed in aldosterone-treated mice, as compared with placebo-treated controls [3]. On the contrary, a modulation of the RAAS by ACE inhibitor therapy induced an increase in Treg cells and decreases in the Th1/Th2 cytokine ratio and pro-inflammatory cytokine secretion in patients with chronic heart failure [7]. 

Myxomatous mitral valve disease (MMVD) is a very frequently acquired cardiac disease in small and medium-sized dogs [8]. This heart disease is responsible for congestive heart failure (CHF), with the onset of cough, polypnea/dyspnea, and pulmonary edema. In dogs, the activation of RAAS during CHF due to MMVD is well-documented, and, for this reason, similarly to humans, these animals are treated with ACE inhibitors and anti-aldosterone diuretic agents, such as spironolactone, to control symptoms and prevent ventricular remodeling [9]. The involvement of the immune system and a pro-inflammatory cytokine response in mitral canine patients with CHF has not yet been extensively investigated. In this study, we selected three cytokines (TNF-α, IL-1β, and IL-6) whose roles in heart diseases have been widely accepted.

TNF-α is the most studied pro-inflammatory cytokine both in experimental models of heart failure and in the course of the natural disease. Dunlay and colleagues [10] reported a correlation between the serum levels of this inflammatory mediator and the mortality rate in humans. TNF-α shows a negative inotropic action on cardiomyocytes by altering calcium homeostasis and promoting apoptosis [11]. It also affects macrophages by promoting the production of other multifunctional cytokines, thus amplifying its action [12]. 

In addition to having a negative inotropic and pro-apoptotic action similar to TNF-α, IL-1 reduces systolic function by suppressing the stimulation of β-adrenergic receptors [1] and activates the fibroblasts which results in myocardial fibrosis [13].

IL-6 acts on the various cell types that are involved in heart failure. It appears that its anti- and pro-inflammatory actions largely depend on the activated signaling pathways. The classic signaling is associated with an anti-inflammatory action, while the alternative pathway (called “trans-signaling”) is responsible for pro-inflammatory mechanisms [1]. IL-6 promotes a hypertrophic response in cardiomyocytes and the proliferation of fibroblasts, suggesting an important role in adverse myocardial remodeling [14,15].

Camacho et al. [16] found a significant increase in IL-1β in dogs with mitral disease, as well as a close correlation with the diameters of the atrium and left ventricle, which increase as the disease progresses. 

Fonfara et al. [17] found higher blood levels of leptin mRNA in dogs with CHF due to acquired heart disease as compared to both dogs with congenital heart disease and healthy dogs. Similarly, myocardial leptin expression was increased in dogs with CHF due to the acquired heart disease compared to dogs with congenital abnormalities and healthy dogs [17], with higher expression in dogs undergoing advanced CHF compared to those in the early and intermediate stages of CHF. Finally, Kim et al. [18] found higher serum leptin levels in dogs with advanced MMVD than in healthy animals, but not in those patients in the early or intermediate stage of MMVD. The same authors found no differences regarding IL-18 between cardiopathic and healthy dogs.

The aim of this study was to verify the existence of a pro-inflammatory condition and a dysfunction of the immune system in dogs with MMVD in different stages of severity through the blood assessment of Treg cells, leptin, and three pro-inflammatory cytokines. Immunological and pro-inflammatory profiles were related to the stage of MMVD and to echocardiographic parameters indicative of cardiac remodeling.

## 2. Materials and Methods

### 2.1. Animals and Ethical Statement

A total of 36 dogs, 14 females (11 spayed) and 22 males (3 neutered) affected by MMVD were enrolled in the study at the Veterinary Educational Hospital, Department of Veterinary Medicine and Animal Productions (University of Naples Federico II). Mean age and body weight (BW) at enrollment were, respectively, 10.7 ± 2.77 years (age ranged between 5 and 14 years) and 10.9 ± 6.69 kg (BW ranged between 3.1 and 30 kg). The mean body condition score (BCS) was 5.0 ± 0.75 (BCS ranged between 4 and 6). Regarding dog breeds, the enrolled dogs included 16 mixed-breed dogs, 7 Cavalier King Charles spaniels, 4 poodles, 2 Maltese, 1 whippet, 1 Shih-Tzu, 1 bichon frisé, 1 cocker spaniel, 1 dachshund, 1 chihuahua, and 1 border collie. 

Exclusion criteria were concomitant endocrine, infectious or inflammatory conditions, severe hepatic or renal failure, and other concomitant cardiac diseases. Animals that were under pharmacological treatment contemplated for reasons other than MMVD were ruled out from the study. 

Clinical examination, BCS, and bodyweight evaluation were determined by the same operator (LC). Additionally, electrocardiography (ECG), echocardiography, and arterial blood pressure measurements were performed. In addition, serum leptin and cytokines (TNF-α, Il-1β, and IL-6) and blood immunophenotype (CD3^+^ CD4^+^, CD3^+^ CD8^+^ T cells, CD4/CD8 ratio, CD21^+^ B cells, and Treg cells) were measured. 

For the comparison of the inflammatory and immunological parameters, two groups of healthy dogs from a client-owned referral population were established. The first consisted of young animals (n. 11; 6 females and 5 males) and was designated as the control group of healthy young dogs, referred to here as the CTR 1 group. Their average age and weight were 4.1 ± 0.82 years and 13.8 ± 4.30 kg, respectively. The second group consisted of elderly dogs (n. 12; 6 females and 6 males) and was designated as the control group of healthy elderly dogs, referred to here as the CTR 2 group. Their average age and weight were 9.6 ± 0.98 years and 14.8 ± 6.15 kg, respectively. Regarding dog breeds, the CTR 1 group included only mixed-breed dogs, while the CRT 2 group included 11 mixed-breed dogs and 1 cocker spaniel. The mean BCS of the CTR 1 group was 4.8 ± 0.6, while that of the CTR 2 group was 5.2 ± 0.75. Based on their clinical examinations and cardiovascular assessments, as well as the complete blood counts (CBC) and biochemical profiles, all of the control dogs were confirmed to be clinically healthy.

All clinical and instrumental procedures, such as the collection of samples for the analysis used in this study, were approved by the Ethics Committee of the University of Naples Federico II (OPBA, CSV, University of Naples Federico II, PG/2022/0018539), following local and national laws, regulations, and guidelines. The study did not cause any discomfort for the animals and was conducted with correct clinical management.

### 2.2. Electrocardiography, Echocardiography, and MMVD Classification

A standard 6-lead electrocardiogram (ECG model 08SD, BTL Italia, Salerno, Italy) was obtained with animals in right lateral recumbency (paper speed, 50 mm/s; calibration at 1 mV = 1 cm). A complete, conventional echocardiographic examination was obtained following the standardized methodologies [19,20,21] by the same operator (DP). For each parameter, the average value obtained from three consecutive measurements was considered. In particular, M-mode measurements were represented by left ventricle (LV) internal diameter at end diastole (LVIDd) and at end systole (LVIDs). LVIDd was indexed by allometric scaling based on body weight [22]. LV end-diastolic volume (EDV) and LV end-systolic volume (ESV) [EDV = (LVIDd^3^ × 7)/(LVIDd + 2.4); ESV = (LVIDs^3^ × 7)/(LVIDs + 2.4)] were obtained using the Teicholz formula. End-diastolic volume index (EDVI) (normal value, <100 mL/m^2^) and end-systolic volume index (ESVI) (normal value, <30 mL/m^2^) were calculated dividing LV volumes by body surface area. The 2D short-axis view at the base of the heart was used to calculate the left atrium/aorta diameter ratio (LA/AO). The echocardiographic diagnosis of MMVD was made on the basis of the evidence of the prolapse or thickening of one or both mitral valve leaflets using the 2D, right parasternal, long-axis view and the assessment of a regurgitant jet on the left apical 4-chamber view with the echo-color Doppler technique [23].

The studied population of dogs with MMVD was classified according to the American College of Veterinary Internal Medicine (ACVIM), which includes four stages of disease severity [24]:

A. Dogs belonging to breeds at risk for MMVD that have no evident cardiac abnormalities. 

B1. Dogs with MMVD that have no clinical signs and no evidence of cardiac remodeling.

B2. Dogs with MMVD that have no clinical signs but show cardiac remodeling due to the volumetric overload of the left side of the heart. 

C. Dogs with MMVD and past or current clinical signs of heart failure associated with volumetric overload. 

D. Dogs with end-stage MMVD and clinical signs of heart failure unresponsive to standard treatment. 

### 2.3. Blood Sample Collection

A CBC, serum biochemical panel, leptin, cytokines (TNF- α, IL-1 β, and IL-6), and an immunophenotypic evaluation (CD3^+^ CD4^+^, CD3^+^ CD8^+^ T cells, CD4:CD8 ratio, Treg cells) were performed. The blood sampling did not cause any discomfort or stress to the enrolled dogs, as required by national legislation. Ethics committee approval was obtained (see ethics statement). A quantity of 10 ml of blood was collected by jugular venipuncture for each dog, which had been kept fasting for 12 h. Each blood sample was split into 3 tubes. Within 30 min of collection, a tube with potassium ethylenediaminetetraacetic acid (EDTA) was used for the CBC; for the immunoassays, a tube with EDTA was used and stored at room temperature for up to 5–6 h before testing; a third tube without anticoagulant was allowed to clot and centrifuged at 908× *g* for 15 min at 4 °C to obtain blood serum. The serum was subsequently stored at −80 °C and thawed immediately prior to evaluation of the biochemical profile and the leptin and cytokine assay.

### 2.4. CBC and Serum Biochemistry

A semi-automatic cell counter (IDEXX, ProCyte Dx, Westbrook, ME, USA) was used for the CBC. In order to measure urea, creatinine, glucose, triglycerides (TG), total cholesterol (T-Chol), alanine aminotransferase (ALT), alkaline phosphate (ALP), total bilirubin (T-Bil), sodium, potassium, albumin and total protein (TP), a semi-automatic chemical chemistry analyzer was used (OLOT, Spinreact, Girona, Spain). 

### 2.5. Leptin and Inflammatory Cytokines Assay

Serum leptin, TNF-α, IL-1β, and IL-6 assays were performed using a canine cytokine ELISA kit (Cloud-Clone Corp., Katy, TX, USA). The detection limit of serum leptin was 0.112 ng/mL, and the range test was 0.312–20 ng/mL. The detection ranges for TNF-α, IL-1β, and IL-6 were 15.6–1000 pg/mL, 7.8–500 pg/mL, and 15.6–1000 pg/mL, respectively. For all of the ELISA analyses, the intra- and interassay coefficient of variability (CV) were <10 and <12%, respectively. Absorbance was assessed at 450 nm with a microplate reader (GDV DV 990BV4 programmable MPT reader, Agilent Technologies, Santa Clara, CA, USA).

### 2.6. Monoclonar Antibodies, Immunofluorescence, and Flow Cytometry

The immunophenotypic study was conducted with the previously described approaches [25,26,27,28,29]. To study the immunological profiles of the enrolled dogs, peripheral blood mononuclear cells (PBMCs) were used. Immune-fluorescence techniques, combined with flow cytometry, were employed for the evaluation of CD3^+^, CD3^+^ CD4^+^, CD3^+^ CD8^+^ T lymphocytes and CD4^+^ CD25^high^ Foxp3^+^ regulatory T cells (T_reg_). FITC, Phycoerythrin (PE), Cy-chrome and Allophycocyanin (APC)-labeled monoclonal antibodies (mAbs) against dog CD3 (Clone CA17.2A12), CD4 (Clone YKIX302.9), CD8 (Clone YCATE55.9), CD45 (clone CA12.10C12), and isotype-matched controls were used (Serotec Ltd., London, UK). A combination of dog-specific anti-CD3 and anti-CD4 or anti-CD8 mAbs was employed to analyze the CD8^+^ and CD4^+^ T cells.

The identification of Treg lymphocytes was based on the Foxp3 expression. Cells were subjected to fixation and permeabilization using a commercial kit (FoxP3 Staining Set, eBioscience). Cross-reactive anti-FoxP3 mouse mAb (Clone FJK-16 s, eBioscience, San Diego, CA, USA) was used to detect Foxp3-expressing CD4^+^ T cells. A FACScalibur apparatus and CellQuest analysis software (Becton Dickinson, Mountain View, CA, USA) were used for flow cytometry and data analysis approach.

### 2.7. Statistical Analysis

Mann–Whitney or Wilcoxon paired signed-rank tests were employed for statistical analysis (InStat version 3.0, GraphPad Software Inc., San Diego, CA, USA), as indicated in the figure legends. Correlations were analyzed using Pearson’s correlation. Results were considered significant at *p* < 0.05. 

## 3. Results

### 3.1. Clinical Classification, Echocardiographic Parameters, CBC, and Serum Biochemistry

According to the ACVIM classification, 12 dogs were found to be in stage B1, 11 dogs in stage B2, 10 in stage C, and 3 in stage D. 

Regarding treatment at inclusion, all dogs in stage B1 were neither symptomatic nor received drugs. In stage B2, all dogs were asymptomatic, and only 4 dogs were not administered drugs at the time of presentation; 5 were being treated with pimobendane and 3 with an angiotensin-converting enzyme inhibitor. In stage C, 5 dogs were not administered drugs at the time of presentation with clinical signs of CHF. The remaining dogs were in clinical heart failure, and 6 of them were being treated with pimobendane, 3 with an angiotensin-converting enzyme inhibitor, and 6 with furosemide. In stage D, 3 dogs were being treated with pimobendan, 3 with an angiotensin-converting enzyme inhibitor, 2 with spironolactone, 3 with torasemide, 1 with sildenafil, and 1 with mexiletine.

**Figure 1:**
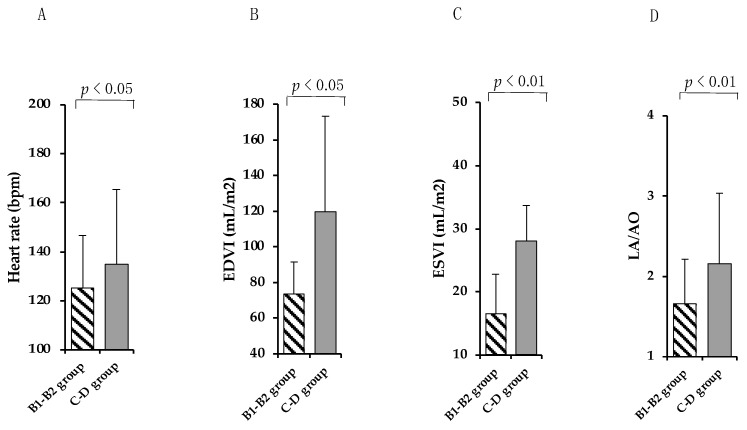
**The echocardiographic examination of enrolled dogs.** Heart rate (**A**), EDVI (**B**), ESVI (**C**), and LA/AO ratio (**D**) in the mild and moderate MMVD (B1–B2) group (dashed column) and in the severe and very serious MMVD (C–D) group of dogs (grey column). Wilcoxon’s matched-pairs signed-rank test was performed to compare the different groups. NS means “no significant difference”.

Upon ECG analysis, all dogs showed a sinus rhythm except one that was in stage D and had numerous left-ventricular extrasystoles. The heart rate was significantly higher (*p* < 0.05) in the C–D group (148 ± 27 heart beats per minute (bpm)) than in the B1–B2 group (125 ± 21 bpm) (Figure 1A). Upon echocardiographic examination of the MMVD dogs end-diastolic volume index (EDVI) (*p* < 0.05) and end-systolic volume index (ESVI) (*p* < 0.01) resulted in being significantly increased in the C–D group (EDVI: 152.8 ± 42.09 mL/m^2^; ESVI: 33.6 ± 19.53 mL/m^2^) compared to that of the B1–B2 group (EDVI: 73.4 ± 18.33 mL/m^2^; ESVI: 16.5 ± 8.88 mL/m^2^) (Figure 1B,C), according to the stage of severity of the disease. Furthermore, a significant increase (*p* < 0.01) in the LA/AO ratio (Figure 1D) was observed in the C–D group (2.5 ± 0.33) compared to the B1–B2 group (1.64 ± 0.29). 

**Figure 2:**
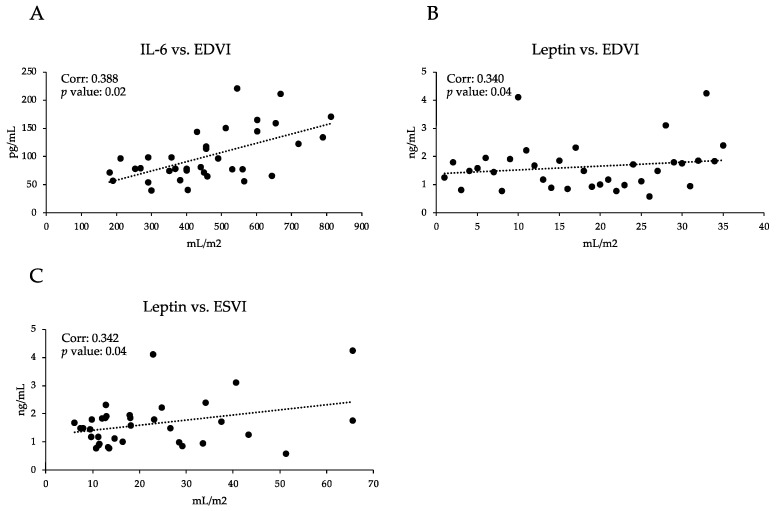
**Pearson’s correlation.** Leptin (ng/mL) vs. EDVI (mL/m^2^) (**B**); Leptin vs. ESVI (mL/m^2^) (**C**); and IL-6 (pg/mL) vs. EDVI (**A**) of the mild and moderate MMVD (B1–B2) group and the severe and very serious MMVD (C–D) group of dogs.

A significant positive correlation (*p* < 0.05) was found for the serum IL-6 level with EDVI (Figure 2A) in dogs with MMVD. Furthermore, a significant positive correlation (*p* < 0.05) was found for the serum leptin level with both EDVI and ESVI (*p* < 0.05) (Figure 2B,C). 

Regarding the CBC and biochemical parameters, there were no statistically significant differences between the groups (data not shown).

### 3.2. Immune Profile

The immunological data of the dogs with MMVD were evaluated by comparison with the immune profile of the healthy young (CTR 1) group and the healthy elderly (CTR 2) group.

**Figure 3:**
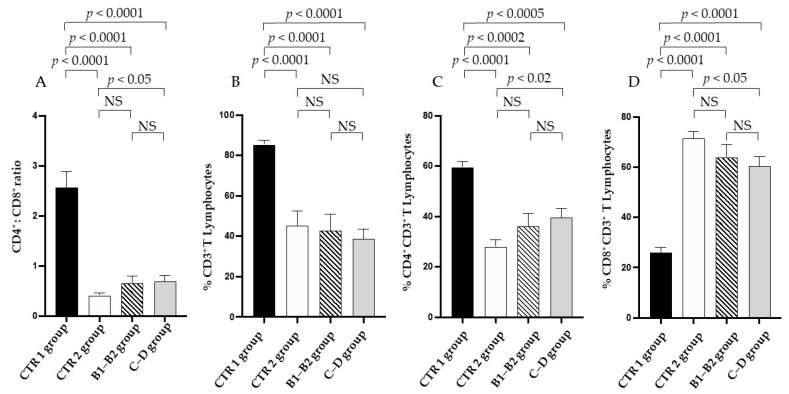
**The immune profile of enrolled dogs.** CD4:CD8 ratio (**A**), CD3^+^ T (**B**), CD4^+^ CD3^+^ (**C**), and CD8^+^ CD3^+^ (**D**) lymphocytes in the healthy young (CTR 1) group (black column), the healthy elderly (CTR 2) group (white column), the mild and moderate MMVD (B1–B2) group (dashed column), and the severe and very serious MMVD (C–D) group (grey column). A Mann–Whitney matched-pairs signed-rank test was performed to compare the different groups. NS means “no significant difference”.

The CTR 2, B1–B2, and C–D groups showed an inverted CD4:CD8 T cell ratio when compared to the CTR 1 group (Figure 3A). The CD4:CD8 T cell ratio significantly increased in the C–D group when compared to the CTR 2 group (Figure 3A). The CD3^+^ T cell percentage significantly decreased in both the healthy (CTR 2) group and the cardiopathic (B1–B2 and C–D) groups of elderly dogs when compared with young dogs (CTR 1) (Figure 3B). Intriguingly, the CD4^+^ CD3^+^ T percentage decreased, while the CD8^+^ CD3^+^ T increased in the CTR 2, B1–B2, and C–D groups when compared to the CTR 1 group (Figure 3C,D, respectively).

The B1–B2 and C–D groups presented no change in the percentage of CD3^+^ T lymphocytes when compared to the CTR 2 group (Figure 3B). 

In addition, the CD4^+^ CD3^+^ T cell percentage in the C–D group was significantly higher than in the CTR 2 group (Figure 3C). Conversely, the CD8^+^ CD3^+^ T cell percentage in that same patient group was lower than in the CTR 2 group (Figure 3D).

**Figure 4:**
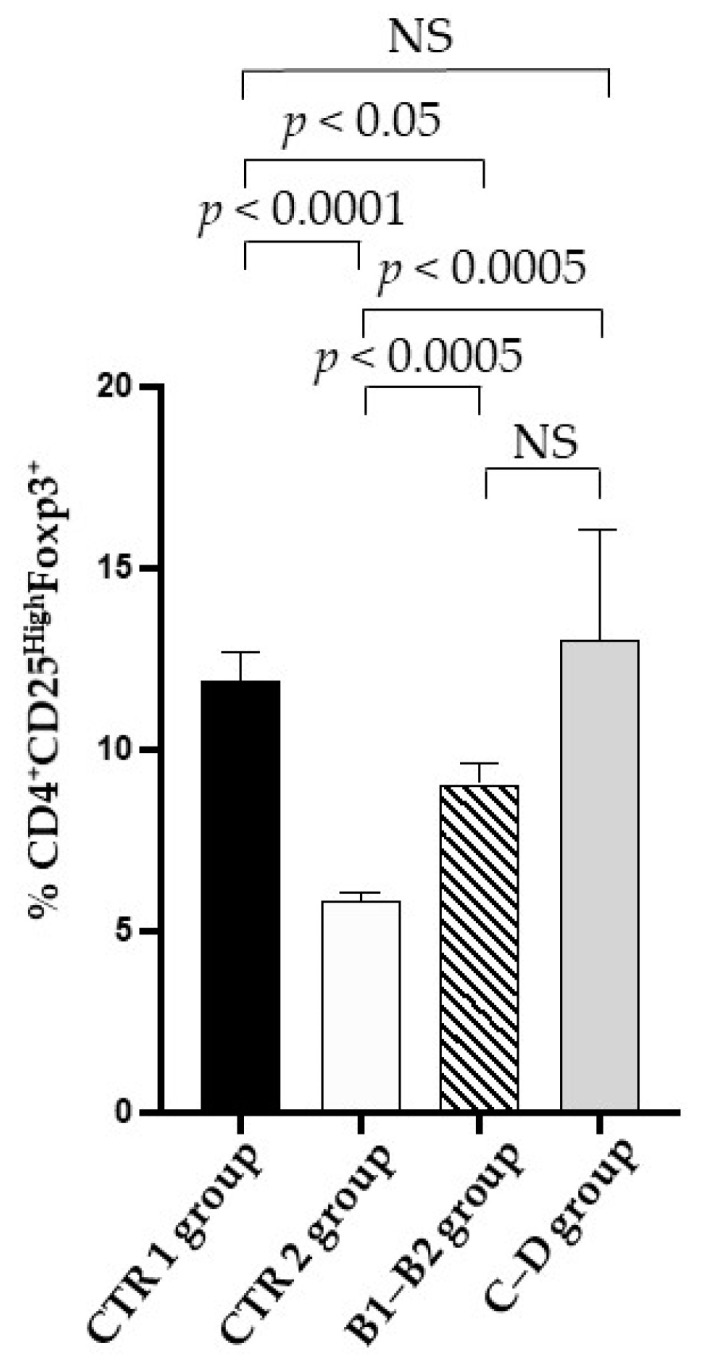
**Treg cells in enrolled dogs.** Treg percentage (CD4+ CD25 high FoxP3^+^ T cells on *y* axis) in the healthy young (CTR 1) group (black column), the healthy elderly (CTR 2) group (white column), the mild and moderate MMVD (B1–B2) group (dashed column), and the severe and very serious MMVD (C–D) group (grey column). A Mann–Whitney matched-pairs signed-rank test was performed to compare the different groups. NS means “no significant difference”.

Furthermore, a significant decrease in the Treg percentage was observed in the CTR 2 group when compared to the CTR 1 group (Figure 4). The Treg percentage was observed to be significantly increased in the B1–B2 and C-–D groups when compared to the CTR 2 group (Figure 4). No statistical significance was evident between the CTR 1 group and the C–D group. Therefore, it is possible to assume that the Treg percentage in the C–D group was similar to that of the CTR 1 group (Figure 4).

### 3.3. Pro-Inflammatory Profile

**Figure 5:**
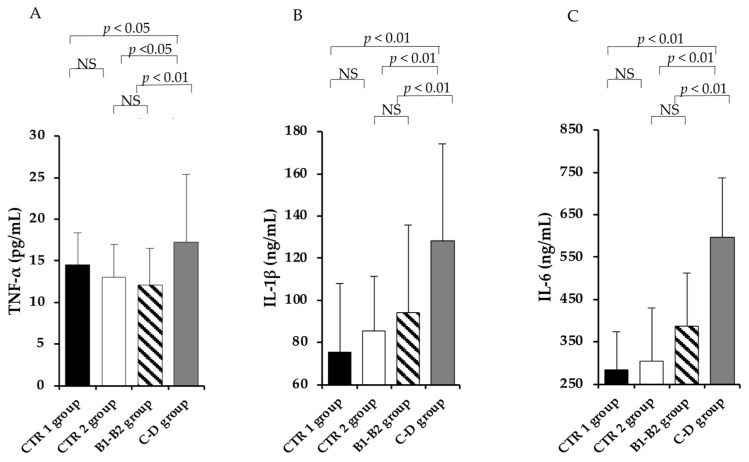
**The inflammatory cytokines in enrolled dogs.** TNF-α (**A**), IL-1 β (**B**), and IL-6 (**C**) in the healthy young (CTR 1) group (black column), the healthy elderly (CTR 2) group (white column), the mild and moderate MMVD (B1–B2) group (dashed column), and the severe and very serious MMVD (C–D) group (grey column). Wilcoxon’s matched-pairs signed-rank test was performed to compare the different groups. NS means “no significant difference”.

The level of TNF-α (Figure 5A) was higher in the C–D group compared to the B1–B2 group (*p* < 0.01), and such a difference was also evident when comparing the CTR groups with the C–D group (*p* < 0.05). Levels of IL-1β and IL-6 (Figure 5B,C, respectively) showed a similar trend, resulting in significantly higher levels (*p* < 0.01) in the C–D group when compared with both the B1–B2 group and the 2 CTR groups. No differences were registered as occurring between CTR groups 1 and 2 when compared with the B1–B2 group. Comparisons between the mild and moderate MMVD dogs and the severe MMVD dogs, and also those between the CTR dogs and the MMVD dogs, showed no differences in leptin levels.

## 4. Discussion

To the best of our knowledge, this is the first study to investigate changes in blood pro-inflammatory cytokines and immunophenotypes in dogs affected by MMVD at different stages of heart failure. In this study, MMVD was diagnosed based on a complete cardiovascular assessment and classified according to the ACVIM guidelines.

Focusing on the canine immunological asset [30] related to total CD3^+^ T lymphocytes and on the 2 main T-cell subpopulations: CD4^+^ CD3^+^ T and CD8^+^ CD3^+^ T lymphocytes [30,31], we observed that the percentage of CD3^+^ T cells was lower in the healthy elderly dogs (CTR 2) and the cardiopathic (B1–B2 and C–D) groups of dogs when compared with the young dogs (CTR 1). Moreover, the immunological profile evaluation highlighted that all of the elderly dogs enrolled in the study, both as healthy controls (CTR 2) and as MMVD patients (B1–B2 and C–D groups), showed a reversed CD4:CD8 T cell ratio when compared to the healthy young control dogs (CTR 1). This result agrees with the literature that reports an unbalanced CD4: CD8 T cell ratio in older dogs and a decline in immune assets with aging in dogs [31]. These data appear interesting as dogs presenting comorbidities with the condition of MMVD were excluded from the study and, notably, the aforementioned alterations in the immune profile were also evident in healthy elderly dogs. Therefore, excluding the interference of possible comorbidities or other diseases, it is likely that we can ascribe the immune-profile alterations to the older age of the dogs [31,32,33]. In addition, according to previous observations [32,33], we noted that the reversed CD4:CD8 ratio appeared to be due to the percentage reduction in CD4^+^ CD3^+^ T cells and to the increase in CD8^+^ CD3^+^ T cells in all elderly dogs. 

However, the CD4:CD8 T cell ratio tends to increase in dogs with more severe MMVD (the C–D group) and becomes significantly different and higher than in the healthy elderly control dogs (CTR 2). In this regard, the rebalancing of the ratio seems to depend on the occurrence of the increase in the percentage of CD4^+^ CD3^+^ T cells and of the decrease in CD8^+^ CD3^+^ T cells in dogs with more severe disease (the C–D group) when compared to healthy elderly control dogs (CTR 2). 

Therefore, in sick MMVD dogs, although an increase in CD8^+^ CD3^+^ T cells typical of elderly dogs was observed, the percentage of CD4^+^ CD3^+^ T lymphocytes was higher when the disease was more severe (the C–D group) and when compared with healthy elderly dogs (CTR 2). This finding suggests that a pro-inflammatory T-cell response [25,26,28,30,31] could be active in the most severe stages of MMVD. It is of note that dogs with less severe pathology (the B1–B2 group) also showed a tendency toward increased levels of CD4^+^ CD3^+^ T cells, even if not statistically significant. Since Th cells are responsible for cytokine production [25,26,28], this occurrence could lead to or at least foster a cytokine production increase in the MMVD stages.

Of particular interest appears to be the increase in Treg cells [4,6] observed in our cohort of MMVD dogs. Treg cells are involved in the maintenance of peripheral tolerance [4,5,6] and in etiopathogenetic and pathophysiological mechanisms in the dog [25,26,27,28,29]. In our cohort, the B1–B2 and C–D groups expressed a significantly higher percentage of Treg than did the healthy elderly dogs, which was comparable to young dogs. This finding could suggest the occurrence of a homeostatic mechanism aimed at promoting immunoregulation [25,26,27,28,29], reducing the potential pathophysiological mechanisms, and immune-mediated tissue damage in the heart. In this regard, the increase in Tregs may be necessary to contain the here-observed increase in T helper (Th) cells, predominantly with the CD4^+^ CD3^+^ T cell phenotype [25,26,27,28,29], and the hyperproduction of TNF-α, Il-1β, and IL-6 in the more severe stage of canine MMVD. It is also interesting to note that, although experimental evidence suggests that aldosterone decreases Treg cells, Shao and colleagues [34] showed an upregulation of Treg cells, associated with the production of TGF-β and myocardial fibrosis in human patients with advanced CHF. In particular, there is much evidence from studies performed on mouse models, human and canine mitral valve samples, and cell cultures that supports the role played by TGF-β upregulation in the onset and progression of MMVD. In fact, TGF-β triggers the endothelial-to-mesenchymal transition, which in turn leads to the phenotypic differentiation of the valve interstitial cells (VICs) in their active form, which are ultimately responsible for the degrading of the extracellular matrix (ECM) which is expressed with the typical morphostructural changes. It is noteworthy to underline that the activation of VICs is also associated with an increase in Il-1β production, which, in turn, negatively affects the ECM [35]. 

In the canine species, the evidence of cytokine activation during CHF is still minimal and conflicting. In dogs, CHF is mainly due to chronic mitral valve disease (MMVD), characterized by myxomatous degeneration. Generally, the disease is more prevalent in small-sized dogs, and in some small breeds, it is reported to have an incidence of close to 100% over the lifetime, but large-sized dogs can be affected as well [23].

It is known that cytokines are involved in several biological mechanisms and exert a pivotal role in inflammation and immunology. Indeed, components involved into the inflammatory progression contribute to heart failure and negatively influence cardiac homeostasis [36]. Numerous experimental and clinical studies on humans have suggested an incisive role for inflammatory cytokines, both in acute and chronic heart failure, regarding the development of the adverse cardiac remodeling that is responsible for systolic and diastolic dysfunction [37]. Regardless of the different etiological factors, the activation of the inflammatory cascade, both locally and systemically, represents a consistent feature of heart failure, and human patients have shown elevated serum cytokine levels [38]. The cytokine activation has a protective significance (e.g., IL-6 preserves mitochondrial function and IL-1 stimulates a compensatory hypertrophy), but, when it is excessive, it may have deleterious effects on failing hearts. For this reason, blocking the upregulation of inflammatory cytokine signaling could represent a new therapeutic approach for cardiopathic patients [1].

In our study, TNF-α, IL-1β, and IL-6 significantly increased according to the severity of the disease. As reported by Oral et al. [39], in the symptomatic and asymptomatic stages of MMVD in humans, high levels of TNF-α have been observed in blood, whereas the data reported in literature for canine species are controversial. In our study, we found a significant increase of TNF-α when comparing CTR dogs with severe MMVD dogs, and similar results were described by Freeman et al. [40]. On the other hand, our results contrast with those of Zois et al. [41], in which TNF-α and IL-6 levels were analyzed in 68 dogs, classified into 5 groups according to the degree of mitral regurgitation and the presence of decompensated CHF, but the TNF-α level could not be quantified, and the IL-6 level was quantifiable only in <25% of the recruited dogs. Mavropoulou et al. [42] reported no differences for the peripheral cytokines, IL-1β, IL-6, and TNF-α, in dogs affected by MMVD (at different stages: B1, B2, and C) when compared with healthy dogs. Also, IL-1β, IL-10, and TNF-α levels were not quantifiable in >60% of the MMVD dogs’ serum by Kim et al. [18]. Fonfara et al. [17] reported a significant increase of IL-1β in blood from dogs with CHF, while no differences in IL-6 were registered between healthy dogs and affected dogs. The main shortcoming of these studies was that, for a high number of dogs, the evaluated cytokines were unquantifiable in the serum samples, probably due to the high variability of these parameters and/or of the analytical methods used for detection, making it difficult to draw significant conclusions. More recently, in a small number of dogs with MMVD, Camacho et al. [16] found a progressive and significant increase in the serum concentrations of IL-1β in stage B2 and stage C, with a moderate correlation between the cytokine level and the LVIDd and LA/AO. Also, Svete and colleagues [43] reported a concentration of TNF-α significantly higher in dogs in CHF compared to those not in CHF when considering a group of patients with MMVD and dilated cardiomyopathy; another study failed to show differences in IL-6, TNF-α, and several other cytokines in the different ACVIM stages, without correlation with the echocardiographic parameters suggestive of cardiac remodeling [44]. 

Concerning leptin, an increase of serum leptin concentration has been proposed in humans as a risk factor for cardiac disease [45]. In our research, although a correlation was found between leptin level and the EDVI and ESVI of MMVD dogs, no differences were registered for leptin levels between the CTR dogs and the MMVD dogs. In contrast, Kim et al. [18] reported significantly different levels in healthy dogs and dogs affected by MMVD, but only to a severe degree, thus suggesting an association between circulating leptin levels and the severity of cardiac disease.

## 5. Conclusions

Overall, our results show a significant increase in cytokine levels in dogs with more advanced MMVD and therefore with greater hemodynamic impairment, and the relative increase in Treg cells could represent a regulatory mechanism that limits the inflammatory immune response. Furthermore, the positive correlation between IL-6 and the LV diastolic volume suggests that inflammatory activation may play a role in the ventricular remodeling associated with the progressive volumetric overload in MMVD; however, further documentation is required.

## 6. Limitations

One important limitation of this cross-sectional study was the limited number of dogs with MMVD included, so patients were grouped into only two groups for statistical purposes. Therefore, this should be considered a pilot study, and further studies should be performed on a greater number of subjects, stratified into the different individual stages of the ACVIM classification.

## Data Availability

The raw data supporting the conclusions of this study will be made available by the authors on request.
